# Oral Health Knowledge and Habits of Hungarian Monozygotic and Dizygotic Twins: A Pilot Study

**DOI:** 10.1016/j.identj.2023.06.012

**Published:** 2023-07-21

**Authors:** Klaudia Liptak, Laura Liptak, Noemi Katinka Rozsa, Peter Hermann, Adam Domonkos Tarnoki, David Laszlo Tarnoki, Daniel Vegh

**Affiliations:** a Department of Prosthodontics, Semmelweis University, Budapest, Hungary; b Department of Paediatric Dentistry and Orthodontics, Semmelweis University, Budapest, Hungary; c Medical Imaging Centre, Semmelweis University, Budapest, Hungary

**Keywords:** Monozygotic twin, Dizygotic twin, Oral health, Advocacy, Health education, Public health

## Abstract

**Objective:**

The aim of this research was to collate and analyse the data on the oral health knowledge and the related habits of a Hungarian cohort of monozygotic (MZ) and dizygotic (DZ) twins using the newly developed World Health Organisation Oral Health Questionnaire for Adults (Annex 7).

**Method:**

A total of 15 sets of MZ twins and 14 sets of DZ twins (58 individuals) aged between 18 and 71 years were enrolled in the study. Each participant had to fill out a web-based questionnaire which comprised 23 questions (Google Forms). The data were collated and the oral health/hygiene habits of MZ and DZ twins were compared.

**Results:**

No significant differences were detected between MZ and DZ twins with regards to their daily tooth-cleaning habits or the tooth-cleaning products used by the 2 groups. For instance, when asked how often they clean their teeth, 80% of MZ twins and 71% of DZ twins responded similarly. Further, both groups provided similar responses when questioned about the use of fluoride toothpaste, frequency of dental visits, and dental counselling received as well as a number of other parameters such as snacking of sweets and fear of visiting dentists.

**Conclusions:**

Our pilot analysis of the questionnaire responses from MZ and DZ twins in Hungary did not indicate any significant differences in their oral care habits in general. Further studies with a large cohort are required to confirm or refute our findings.

## Introduction

The study of human twins has always been fascinating. The interest in the subject has been further enhanced by the phenotypic and behavioural differences between monozygotic (MZ) and dizygotic (DZ) twins.^1^ Genetic and environmental factors are related to the behavioural and other phenotypic attributes of MZ and DZ twin pairs. Since MZ twins share almost identical genomes, any variation between them must stem from environmental influences alone.^2^^,^^3^ Hence, twin studies in general offer valuable insights on the environmental factors affecting their behaviour.

As there are no studies, to our knowledge, on whether there are differences between oral hygiene and risk factors for oral disease between MZ and DZ twins, a pilot study was performed amongst such pairs in Hungary. For this purpose, we used the World Health Organisation (WHO)–recommended simplified structured questionnaire survey (Annex 7).^4^

The data collection was performed using telemedicine technology during the COVID-19 pandemic period. This approach eliminated the need for in-person visits and travel by the interviewees, as well as their travel cost.^5^ In addition, the efficient and effective data collection tools and methodology popularised during the COVID-19 pandemic were of great utility for our survey.^6^

This study, therefore, was aimed at eliciting differential features, if any, between oral health knowledge and self-reported oral hygiene habits of a small cohort of MZ and DZ twins in Budapest, Hungary, and its suburbs. The questionnaire included items on oral health knowledge and self-reported daily oral hygiene habits as per the WHO recommended simplified structured questionnaire.^4^ The null hypothesis for the study was that there is no significant difference between the oral care habits of MZ and DZ twins in Hungary.

## Methods

### Participants

A total of 58 twin Hungarian participants (38 women and 20 men), randomly selected from the Hungarian Twin Registry (HTR) database, were used for the online survey. Participation in the questionnaire survey was voluntary.

The questionnaire survey was performed over a 3-month period from October to December 2022. The instrument used for the survey was the Google Survey interphase available on either a computer, tablet, or a mobile phone device, whilst the language of the questionnaire was Hungarian.

Exclusion criteria included an incomplete questionnaire and more than one sample from the same person through an identical email address. Non-twins, if any, were excluded from the study. In addition, we excluded the questionnaire of those whose co-twin did not fill out the questionnaire. The zygosity of twin pairs was determined by a standardised 7-question self-reported questionnaire that yields nearly 99% accuracy. This questionnaire has been the basis of zygosity assessment in the Danish Twin Registry for over half a century.^7^^,^^8^

The study was carried out as per the Declaration of Helsinki and approved by the Semmelweis University Ethical Board (ethical clearance number: SE RKEB 2023).

#### Questionnaire

The questionnaire comprised a total of 23 questions including fundamental questions that are considered to be of basic importance for evaluating national oral health.^4^

#### Online survey

The study protocol was based on the Annex 7 of the WHO survey form.^4^ When filling out the questionnaire, the twin pairs used the same identifier, a combination of 3 letters and 3 numbers (eg, ABC123), which the twin pairs proposed. This identifier helped us in the comparison of twin pairs and also to maintain the anonymity of the twin pairs related to their MZ or DZ nature until the code was broken.

#### Data collection and outcome measures

We collected the data online. All data were stored using Google Surveys and Microsoft Excel.

#### Statistical analysis and visualisation

The data were analysed using an Excel spreadsheet, which was also used to store the data. The significant differences between the 2 groups were tested using Fisher exact test (*P*). Differences below the 5.0% limit (*P* < .05) were considered significant.

### Results

In total, 15 sets of MZ twins (51.7%) and 14 sets of DZ twins (48.3%), a total of 58 individuals, with an average age of 35.2 ± 16.6 years took part in the survey. Most participants resided in Pest County of Budapest (82.8%) and 1.7% in the peri-urban and 17.2% in the rural areas.

In general, the findings showed that there were no significant differences between MZ and DZ twins in their overall general oral health knowledge and the other parameters evaluated. The MZ twins responded similarly to the question of using various teeth-cleaning tools in 27.0% of cases, whilst the DZ twins did so in 21.0% of cases. Two-thirds (67.0%) of participants used manual toothbrushes rather than electric toothbrushes to clean their teeth, and only 12.1% used both types of brushes.

When asked whether they use a fluoride-containing toothpaste, 58.6% of MZ twins replied in the affirmative, whilst 20.7% were ambivalent. Thus, almost one-fifth (20.7%) of the total group were unaware of whether the toothpaste they used contained fluoride.

Overall, 70.0% of both MZ and DZ twins had received oral care advice/education; 67.0% of MZ twins and 64.0% of DZ twins provided similar answers related to oral care education/counselling.

**Fig. 1:**
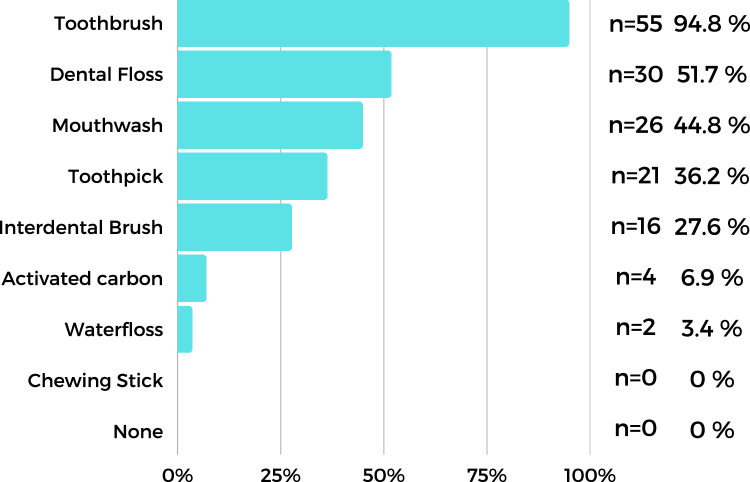
Use of dental cleaning tools by monozygotic and dizygotic twins (cumulative data).

We noted that 80.0% of the identical twin participants and 71.0% of the DZ twins gave the same answer as to how often they brush their teeth. Further, 72.4% of the participants brush their teeth twice or more a day, 27.6% less often. As for the appliance they use for their routine oral hygiene, a vast majority (94.8%) primarily used toothbrushes. Furthermore, 51.7% used dental floss, 44.8% used mouthwash, and 36.2% used toothpicks (Figure 1). Almost 100.0% of MZ twins and 93.0% of DZ twins used a toothbrush.

The relative use of the oral hygiene aids between MZ and DZ twins was also similar and indicted that the toothbrush is the most frequently used cleaning tool (MZ: 73.0%, DZ: 64.0%) followed by flossing (MZ: 53.0%, DZ: 50.0%). In total, 60.0% of MZ and 57.0% of DZ twins gave the same answer on the regular use of dental floss, whilst rates were 80.0% of MZ and 79.0% of DZ twins for the interdental brush; 63.3% of MZ twins and 64.3% of DZ twins use other oral care devices (mouthwash, toothpick, activated carbon, chewing sticks) (Figure 1).

There was no significant difference between MZ and DZ twins (*P* = .355) with regards to the ratio of the use of the main oral care and other toothbrushing utensils (Table 1). Other responses to the questionnaire are as follows. Almost one-half of all the participants (48.3%) had been to the dentist less than 6 months ago (MZ: 27.0%, DZ: 64.0%; *P* > .05), and only a small minority (3.4%) had been 5 years or more. The major reasons for the last visit were routine checkup/treatment (MZ: 43.3%; DZ: 25.0%) and treatment/follow-up (MZ: 36.7%; DZ: 64.3%) (Table 1).

**Table 1:** The response of the monozygotic and dizygotic twin pairs to the World Health Organisation (WHO) recommended simplified structured questionnaire survey.

Question	Response			
	Monozygotic twins, No. (%)		Dizygotic twins, No. (%)	
No. of participating twin pairs	15		14	
During the past 12 months, did have any discomfort in your teeth or mouth ?	9	(60%)	12	(86%)
Do you wear removable dentures?	8	(53%)	10	(71%)
Would you describe the state of your teeth and gums as very good or excellent?				
• Teeth	6	(40%)	6	(43%)
• Gums (gingiva)	6	(40%)	7	(50%)
How often do you clean your teeth?	12	(80%)	10	(71%)
Do you use the following to oral hygiene aids to clean your teeth?				
• Toothbrush	11	(73%)	9	(64%)
• Toothpaste	15	(100%)	14	(100%)
• Fluoride-containing toothpaste	9	(60%)	8	(57%)
Have you ever received oral care education/counselling?	10	(67%)	9	(64%)
How long has it been since you last saw a dentist?	4	(27%)	9	(64%)
What was the reason for your last visit to the dentist?	7	(47%)	7	(50%)
• Routine checkup	6	(43%)	3	(25%)
• Treatment follow-up	5	(37%)	8	(64%)
Because of the state of your teeth or mouth, how often have you experienced any of the following problems during the past 12 months?				
• Difficulty in biting food	9	(60%)	10	(71%)
• Difficulty chewing food	8	(53%)	11	(79%)
• Difficulty with speech/trouble pronouncing words	13	(87%)	14	(100%)
• Dry mouth	11	(73%)	11	(79%)
• Felt embarrassed due to appearance of teeth	10	(67%)	12	(86%)
• Felt tense because of problems with teeth or mouth	10	(67%)	13	(93%)
• Have avoided smiling because of teeth	9	(60%)	13	(93%)
• Had sleep that is often interrupted	9	(60%)	11	(79%)
• Have taken days off work	13	(87%)	13	(93%)
• Had difficulty doing usual activities	13	(87%)	14	(100%)
• Felt less tolerant of spouse or people who are close to you	11	(0%)	13	(93%)
• Have had reduced participation in social activities	10	(67%)	13	(93%)
• Other	6	(40%)	8	(57%)
Do you prefer sugary foods?	7	(47%)	7	(47%)
How often do you use any of the following tobacco products?				
• Cigarettes	12	(80%)	10	(71%)
• Cigars	14	(93%)	12	(86%)
• Pipe	14	(93%)	12	(86%)
• Chewing tobacco	15	(100%)	13	(93%)
• Snuff	15	(100%)	13	(93%)
During the past 30 days, have you consumed alcohol?	8	(53%)	10	(71%)
Are you afraid of dental treatment?	11	(73%)	11	(79%)

Most of the participants (87.9%) had 20 or more natural teeth and only 1.7% had none. Overall, one-half of MZ twins and almost three-quarters (71.0%) of DZ twins did wore dentures. Relatively few participants had removable dentures (8.5%), whilst 17.2% wore fixed prosthodontics.

Based on the subjective assessment the minority of both DZ and MZ twins (40.0%) considered the condition of their teeth and gums as good or excellent; 10.3% rated the condition of their teeth and gums as poor or very poor. For both the MZ and DZ twins, the health of the teeth and mouth was not a major concern when conducting routine daily activities (Table 1). However, over one-half of both MZ and DZ twins complained of difficulty in biting and chewing food.

Other notable grievance in the whole cohort with over one half of both MZ and DZ twins complaining were difficulty with speech, dry mouth, feeling embarrassed about their dentition feeling tense and waking up because of their dental issues, taken time off due to dental issues (Table 1). As regards eating habits, approximately one-half of MZ and DZ twins preferred sugary foods, mostly drinks with sugar-sweetened tea and sweet pies and jams as their go-to sugary treats**.**

We attempted to identify the respondents’ adverse social habits in the final section of the questionnaire. When questioned on alcohol intake, one-half of all respondents had consumed alcohol in the last 30 days (MZ: 53%; DZ: 71%), and most participants never used tobacco products. Further, a majority of both MZ and DZ twins smoked cigarettes (MZ: 80%; DZ: 71%) and other tobacco products.

In summary, there were no significant differences in the responses to any of the oral hygiene attributes questioned between members of MZ and DZ twins for any of the questions, though there were minor variations (Table 1).

## Discussion

As far as we are aware, this is the first study comparing the oral care/hygiene and related habits of MZ and DZ twins. Taken together, the data imply that there were no behavioural changes in the oral hygiene habits between these 2 cohorts, thus confirming our null hypothesis.

The participants for the web-based questionnaire survey were randomly selected from the HTR database, and they resided in the capital Budapest in Hungary and its catchment area. Their urban or suburban residence status does not appear to have influenced the visits to the dentist and oral hygiene habits, as reported in other previous studies.^9^, ^10^, ^11^ In general, approximately one-half of the participants were well educated, graduated from college/university, or had a high school diploma. This may be a reflection of our cohort that was mainly derived from the urban/suburban areas of Hungary and not from underdeveloped rural regions of the country.

In evaluating the data, we found no significant difference between MZ and DZ twins in the frequency of using dental care tools. Almost 100.0% of the twins regularly use toothpaste, and a majority use a manual toothbrush to clean their teeth. More than half of the participants supplement this with dental floss, the regular use of which can reduce interproximal plaque^12^^,^^13^ and reduce periodontal diseases and interproximal caries.^14^ Although electric toothbrushes are more effective in reducing plaque and gingivitis in both the short and long term,^15^ our cohorts predominantly used manual toothbrushes.

A significant genetic role in the development of caries amongst twins has been demonstrated in a previous study by Bretz et al.^16^ Although we could not ascertain the caries status form the survey, only about one-half of the participants used fluoride-containing toothpaste, which effectively prevents tooth decay. The response to the frequency of dental visits was not that satisfactory, as almost one-half of the participants visited a dentist within the preceding 6 months. These imply that the participants require further education on prevention of oral diseases through regular visits to the dentist.

In terms of the functionality of their dentition, most of the participants did not experience difficulty speaking or chewing, and they didn't experience dry mouth,^17^ and almost one-half of the participants consumed fruit on a daily basis. Furthermore, only a minority of MZ twins prefer sugary diets, and this implies that they will be less prone to caries as excessive sugary snacks lead to caries.^18^^,^^19^

Although the harmful health effects of tobacco products are well known,^20^ a majority of both MZ and DZ twins smoked cigarettes and other tobacco products. This implies that tobacco prevention education in the country is inadequate. We also noted that a majority of the cohort feared dental treatment, which emphasises the importance of addressing dental anxiety as well as ensuring patient comfort during dental visits and the need for further public education programmes of contemporary dentistry, which is virtually painless.

One limitation of this pilot study is the subjective assessments by participants of their oral health and general well-being. Further, the small number of participants is also a limitation, and additional larger studies of MZ and DZ twins need to be conducted to verify or refute our pilot findings. Nevertheless, our data should serve as a basis for future workers in this field.

**Fig. 2:**
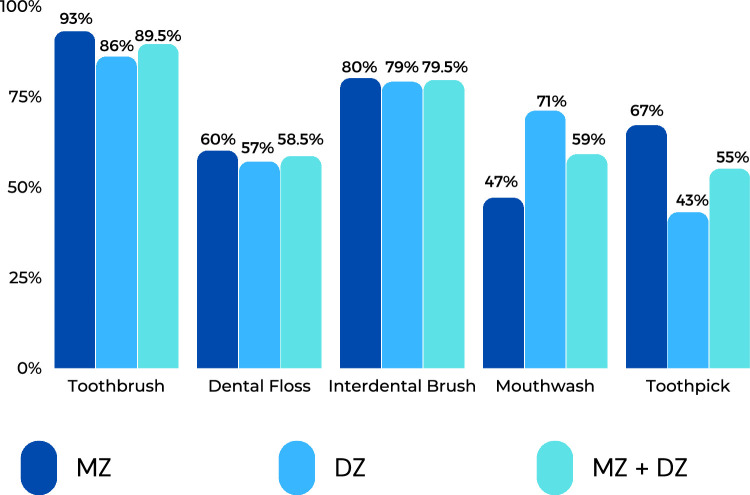
Percentage difference in oral hygiene/care devises used by monozygotic and dizygotic twin pairs.

In conclusion, our study provides important insights into the oral health, hygiene, and related habits of MZ and DZ twins in Hungary. Within the twin population, there appears to be twins who pay more attention to oral care and others who pay less attention. Given the limited sample size and the relatively short questionnaire, our findings suggest that both these groups have a similar degree of oral health habits and awareness. Further research with a larger twin cohort is warranted to confirm or refute our findings (Figure 2).

## Conflict of interest

None disclosed.
